# Phylogenomic diversity of archigregarine apicomplexans

**DOI:** 10.1098/rsob.240141

**Published:** 2024-09-25

**Authors:** Gordon Lax, Eunji Park, Ina Na, Victoria Jacko-Reynolds, Waldan K. Kwong, Chloe S. E. House, Morelia Trznadel, Kevin Wakeman, Brian S. Leander, Patrick Keeling

**Affiliations:** ^1^ Department of Botany, University of British Columbia, Vancouver, Canada; ^2^ Instituto Gulbenkian de Ciência, Oeiras, Portugal; ^3^ Department of Biochemistry and Molecular Biology, University of British Columbia, Vancouver, Canada; ^4^ Institute for the Advancement of Higher Education, Hokkaido University, Sapporo, Hokkaido, Japan; ^5^ Department of Zoology, University of British Columbia, Vancouver, Canada

**Keywords:** multigene, phylogenomics, gregarine, parasite, protist, taxonomy

## Abstract

Gregarines are a large and diverse subgroup of Apicomplexa, a lineage of obligate animal symbionts including pathogens such as *Plasmodium*, the malaria parasite. Unlike *Plasmodium*, however, gregarines are poorly studied, despite the fact that as early-branching apicomplexans they are crucial to our understanding of the origin and evolution of all apicomplexans and their parasitic lifestyle. Exemplifying this, the earliest branch of gregarines, the archigregarines, are particularly poorly studied: around 80 species have been described from marine invertebrates, but almost all of them were assigned to a single genus, *Selenidium*. Most are known only from light micrographs and largely unresolved rDNA phylogenies, where they exhibit a great deal of sequence variation, and fall into four subclades. To resolve the relationships within archigregarines, we sequenced 12 single-cell transcriptomes from species representing all four known subclades, as well as one blastogregarine (which frequently branch with *Selenidium*). A 190-gene phylogenomic tree confirmed four maximally supported individual clades of archigregarines and blastogregarines. These clades are discrete and distantly related, and also correlate with host identity. We propose the establishment of three novel genera of archigregarines to reflect their phylogenetic diversity and host range, and nine novel species isolated from a range of marine invertebrates.

## 1. Background

Apicomplexans are a diverse group of obligate symbionts of animals. Apart from biomedically relevant taxa like *Plasmodium* and *Toxoplasma*, the causative agents of malaria and toxoplasmosis, the vast majority of apicomplexans infect many different invertebrate animal phyla in marine and terrestrial environments, like corals [1], tunicates [2–4], crustaceans [5], annelids [6–8] and insects [9]. Many of these invertebrate parasites are gregarines, an early branching clade of apicomplexans [10,11] that probably occupy a range of symbioses from parasitism to commensalism [12]. Gregarines are very diverse, and the current state of their convoluted taxonomy and phylogeny reflects this [11,13]. A widely accepted view of gregarines classifies them into three distinct groups: eugregarines, blastogregarines and archigregarines [14]. Eugregarines probably represent the majority of gregarine diversity and include species with a very wide range of animal hosts from marine, freshwater and terrestrial environments [9,13,15]. There is evidence that the ‘neogregarines’, another historically common group, should be part of eugregarines based on molecular data [16]. In stark contrast to eugregarines, blastogregarines currently only contain three described species in the genus *Siedleckia* and one in *Chattonaria* [14], which have been found to only infect Orbiniidae, a group of marine polychaetes. The known diversity of archigregarines is somewhat intermediate, with around 80 species, but they are currently virtually monogeneric—the vast majority of species are in the genus *Selenidium*, which contains upwards of 60 described species from a variety of different marine invertebrate hosts [7,8,17–21]. Most of the rest of the described species are nominally in another genus, *Selenidoides*, based on whether merogony was observed or not. The field has largely ignored this because of the dubious nature of this character [7,18], which is borne out by the fact that in small subunit ribosomal DNA (SSU rDNA) phylogenies, several species transferred to *Selenidoides* (*S. axiferens*, *S. hollandei* and *S. mesnili*) do not form a monophyletic group but are instead mixed with species in the genus *Selenidium* as has been proposed by Paskerova *et al.* [7] (see below).

Consequently, *Selenidium* represents a considerable amount of diversity: the various species are morphologically diverse, but this is most obvious at the molecular level, where different species of *Selenidium* can be more distantly related to each other than are entirely different classes of apicomplexans [6,21,22]. Despite recent advances, our understanding of the evolutionary relationships of archigregarines and blastogregarines remains murky at best, largely due to unresolved SSU rDNA phylogenies [7,23]. Four clades of archigregarines (all *Selenidium*) are routinely recovered in SSU phylogenies, but their phylogenetic relationships to each other, and to other gregarines, remain unclear [7,17,21,22]. These four correspond to the hosts they can be found in lineage Ag1 in several polychaete Sedentaria clades, Ag2 in sipunculids (peanut worms), Ag3 in cirratulids (a group of Sedentaria) and Ag4 in terebellids [7](another group of Sedentaria). Crucially, lineage Ag1 contains the type species *Selenidium pendula* Giard 1884, for which SSU rDNA data are available, making Ag1 the ‘true’ *Selenidium*.

While the divergence of the different *Selenidium* clades has been known for several years now, and efforts to further understand these relationships have been made by combining SSU and large subunit (LSU) rDNA phylogenies, this has fallen short of resolving the phylogenetic placement of archigregarines and blastogregarines [7,17]. This is probably due to the fact that archigregarine and blastogregarine species likely diverged a very long time ago, and the fact that the SSU and LSU rDNAs of some archigregarines are quite divergent, which is known to make accurate phylogenetic estimation difficult [11,17,24,25]. Multigene phylogenetics or phylogenomics have become a common way to resolve these difficult relationships. Recent studies using phylogenomics on apicomplexans have included a small number of species from three out of the four archigregarine groups [9,23], but were missing lineage Ag3, and other groups were mostly represented by only a single species. These phylogenies did not clearly resolve any relationships and presented only moderate support at best for any given topology among archigregarines and blastogregarines, and could not address whether the subgroups are each monophyletic.

To improve our understanding of archigregarine and blastogregarine phylogenetic relationships, we isolated single cells of 12 species of archigregarines representing all known four subclades, and an additional blastogregarine, and generated their single-cell transcriptomes. With these transcriptomes, we generated an updated SSU rDNA gene phylogeny to assess their relationship to species with available molecular data, as well as an updated 190-gene phylogenomic tree to provide a more comprehensive sampling of this group for the first time. We propose to transfer three of the four clades of *Selenidium* to three newly established genera *Lunidium, Devanium* and *Metzidium*, to reflect the diversity, phylogenetic relationships and host specificity of this group. We also propose eight new archigregarine species and one blastogregarine species.

## 2. Methods

### 2.1. Sampling, isolation and microscopy

Several host species of polychaetes (Sedentaria) and sipunculids were collected from marine benthic sites in British Columbia (Canada), Hokkaido (Japan) and Curaçao (electronic supplementary material, table S1). Host animals (table 1) were dissected and had their intestines removed, which were opened in sterile 0.2 µm filtered seawater and agitated by rapid pipetting. This slurry was examined for gregarines using a Leica DMIL inverted microscope. Once identified, gregarine cells were then isolated with a glass micropipette, washed 3–6 times in 0.2 µm filtered seawater, and imaged at 200× to 400× magnification with a Sony A7RIII camera. A single cell was then deposited in SmartSeq2 lysis buffer for single-cell transcriptomics [26].

**Table 1.** Isolated archigregarines and blastogregarines and their corresponding hosts, and whether COI host sequences were recovered. An asterisk (*) indicates COI sequence derived from PCR, all others were extracted from transcriptome assemblies using blastx. *Lunidium laculatum*, *L. shako* and *L. proboscidis* were all isolated from the same host individual.

species	host	host COI
*Selenidium validusae*	*Acrocirrus validus*	yes
*Selenidium pherusae* Ph226	Flabelligeridae sp.	yes*
*Selenidium natalis* nov. sp. SEL2980	*Spirobranchus giganteus*	–
*Selenidium capillus* nov. sp. Ph213	Cirratulidae sp.	yes*
*Lunidium melongena*	*Thelepus japonicus*	yes
*Lunidium laculatum* nov. sp. SNEK	*Eupolymnia* sp. (individual 1)	yes
*Lunidium shako* nov. sp. KNOB	*Eupolymnia* sp. (individual 1)	yes
*Lunidium proboscidis* nov. sp. SNEKD	*Eupolymnia* sp. (individual 1)	yes
*Devanium robustum* nov. sp.	*Cirratulus robustus*	yes
*Devanium cincinnus* nov. sp. Ph216	Cirratulidae sp.	yes*
*Metzidium perlucensae* nov. sp. SQU2901	*Phascolosoma perlucens*	yes
*Siedleckia leitoscoloplosis* nov. sp. BL3	*Leitoscoloplos pugettensis*	yes

### 2.2. Single-cell transcriptomics with SmartSeq2 and sequencing

Cells were subjected to two or three freeze/thaw cycles, after which cDNA was generated and amplified following the SmartSeq2 protocol [26], using 22–24 PCR cycles. The resulting cDNA was made into libraries with Illumina Nextera XT or Illumina DNA Prep kits, and sequenced on several Illumina NextSeq (2 × 150 bp paired end) or MiSeq runs (2 × 250 bp or 2 × 300 bp paired end; see electronic supplementary material, table S1). We isolated multiple cells (2–3) for half of the reported morphospecies and co-assembled them (see below).

### 2.3. Transcriptome assembly

Raw reads were subjected to read error correction with rcorrector v. 1.0.4 [27] using default settings. They were then adapter- and quality-trimmed with Trimmomatic v. 0.39 [28] using the TSO, oligo-dT, ISPCR, and transposase sequences and the following settings: ILLUMINACLIP:adapters.fasta:2:30:10 LEADING:5 TRAILING:5 SLIDINGWINDOW:5:16 MINLEN:60. Corrected and trimmed reads were assembled with rnaSPAdes v. 3.15.1 [29] using corrected and trimmed forward, reverse and unpaired Illumina reads. Where possible, we generated co-assemblies from identical cells isolated from the same host using rnaSPAdes (electronic supplementary material, table S1). Protein-coding sequences were predicted from the assemblies using Transdecoder v. 5.5.0 [30]. Mitochondrial cytochrome c oxidase (COI) sequences of the host animals were extracted from the protein-coding sequences with blastx, using publicly available polychaete and sipunculid COI sequences as queries. We conducted PCR using genomic DNA isolated from animal tissue to generate host COI sequences for some samples using primers LCO1490and HCO2198 (table 1), with conditions detailed in a previous study [31].

### 2.4. SSU rDNA phylogenetics

SSU rDNA sequences of archigregarines were extracted from transcriptome assemblies with barrnap v. 0.9 (https://github.com/tseemann/barrnap). To identify apicomplexan SSU rDNA sequences from contaminants, these extracted sequences were then checked against the NCBI nt database via megablast. We generated a comprehensive SSU rDNA dataset of publicly available apicomplexans, biased towards archigregarines and blastogregarines. Extracted sequences identified as apicomplexan were aligned with this dataset using MUSCLE [32] and trimmed using Gblocks [33], as implemented in SeaView v. 5.0.4 [34]. The final trimmed alignment consisted of 115 sequences with 1129 sites (accessions and lengths are listed in electronic supplementary material, table S2). A phylogenetic tree was estimated with RAxML-NG v. 1.1.0 under the GTR+G model and 1000 non-parametric bootstrap replicates [35].

### 2.5. Phylogenomics

To generate a multigene phylogeny, we used a previously published phylogenomic pipeline and 263-gene dataset of eukaryotes, with a comprehensive sampling of apicomplexans [9,23,36]. As part of this pipeline, single-gene trees for each of the 263 genes were generated and checked by hand for obvious contaminant, paralogous or otherwise aberrant sequences, which were then removed from the dataset. Only genes in which at least 40% of all taxa were present were used in the final concatenation, done with SCaFoS v. 1.25 [37].

A final dataset of 190 genes (38 577 sites) and 63 taxa (see electronic supplementary material, table S3 for details) was used to estimate a phylogenomic tree under the LG+C60+F+G model with 1000 Ultrafast bootstraps [38] (UFB) and 200 non-parametric bootstraps under LG+C60+F+G+PMSF [39], using IQTree2 [40]. To test the impact of missing data on our phylogenetic inference, we generated two additional datasets with the same 63 taxa but differing numbers of genes. The 129-gene dataset included all genes that had at least 70% taxon coverage, and the 22-gene dataset with at least 90% coverage across all taxa. Both of these datasets were run in IQTree2 under model LG+C60+F+G and 1000 UFBs.

We also conducted fast-site removal (FSR) and heterotacheous-site removal (HSR) analyses using scripts from PhyloFisher [41]. These scripts each removed 3000 sites at each step from our main dataset until exhaustion, and we estimated a LG+C20+F+G tree from each removal step with 1000 UFBs. The support values for relevant nodes from each step were then mapped against the number of removed sites. To reduce potential coding bias, we recoded our main 190-gene dataset to SR4, using the aa_recoder.py script in PhyloFisher, and ran the resulting matrix in IQTree2 under model GTR+R6+F and 1000 UFB. gGene concordance factor (gCF) and site concordance factor (sCF) analyses were run on our 190-gene dataset under default parameters in IQTree2 [42,43] (http://www.iqtree.org/doc/Concordance-Factor).

## 3. Results

### 3.1. Morphology

#### 3.1.1. *Selenidium capillus* nov. sp.

**Figure 1.**
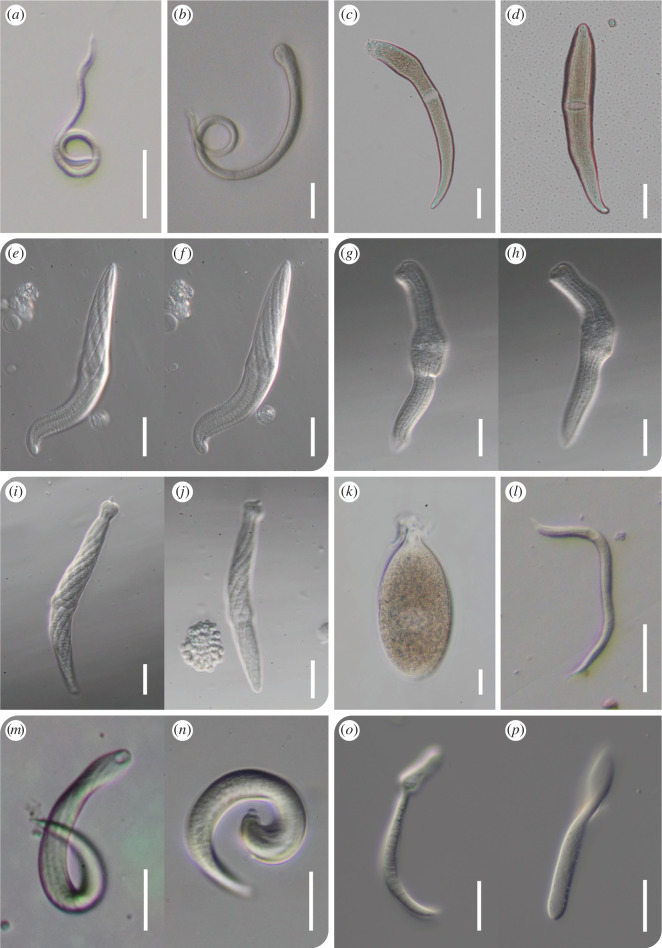
Micrographs of trophozoites of isolated archigregarines and blastogregarines. (*a*) *Selenidium capillus*, isolated cell. (*b*) *Selenidium pherusae* (Ph226), isolated cell. (*c*) *Selenidium natalis*, isolated cell. (*d*) *Metzidium perlucensae*, isolated cell. (*e,f*) *Lunidium laculatum*, same cell, one of three isolated and sequenced cells. (*g,h*) *Lunidium proboscidis*, same cell, one of two isolated and sequenced cells. (*i,j*) *Lunidium shako*, same cell, one of three isolated and sequenced cells. (*k*) *Lunidium melongena*. (*l*) *Devanium cincinnus*, isolated cell. (*m,n*) *Devanium robustum*, different cells from same host. (*o,p*) *Siedleckia leitoscoloplosis*, different cells from same host. Scale bars are 20 µm for all, except 50 µm for A, L and M.

The trophozoite is vermiform, 216 µm long and 14 µm wide, with a pointed anterior end (figure 1*a*). The cell is translucent, and the nucleus is situated around the midpoint of the cell. The cell moved by bending and twisting. Transcriptome data were derived from the cell imaged. Isolated from the intestinal lumen of polychaete worm Cirratulidae sp. (Annelida, Polychaeta, Sedentaria, Terebellida, Cirratulidae), which was collected from subtidal surface sediment (8–11 m below sea level) at Hyacinthe Bay, BC, Canada.

#### 3.1.2. Selenidium pherusae

The trophozoite is vermiform, 212 µm long and 18 µm wide (at its mucron), with a club-shaped mucron (figure 1*b*). A nucleus was not clearly visible, and the cell moved by bending and twisting. Transcriptome data were derived from the cell imaged. Isolated from the intestinal lumen of a bristle-cage worm Flabelligeridae sp. (Annelida, Polychaeta, Sedentaria, Terebellida, Flabelligeridae), which was collected from subtidal surface sediment (8–11 m below sea level) at Hyacinthe Bay, BC, Canada.

#### 3.1.3. *Selenidium natalis* nov. sp.

The trophozoite is >138 µm (cell ruptured during isolation) and 15 µm wide (figure 1*c*). The posterior forms into a pointed end, and the ovoid nucleus is situated around the midpoint of the cell. The cell is light brown in colour. Transcriptome data were derived from the cell imaged. Isolated from intestinal lumen of the Christmas tree worm *Spirobranchus giganteus* (Annelida, Polychaeta, Sedentaria, Sabellida and Serpulidae), which was found in the reef in front of CARMABI research station, Curaçao.

#### 3.1.4. *Metzidium perlucensae* nov. gen. nov. sp.

The trophozoite is vermiform, 117 µm long and 23 µm wide at its widest point, with a pointed posterior end and rounded anterior end (figure 1*d*). Longitudinal folds cover the whole cell, and the elongated nucleus is situated around the midpoint of the cell, closer to the anterior end. The cell appears light brown, and transcriptome data were derived from the cell imaged. Isolated from the intestinal lumen of the peanut worm *Phascolosoma perlucens* Baird, 1868 (Annelida, Sipuncula, Phascolosomatidae), found in the reef in Piscadera Bay, in front of the CARMABI research station, Curaçao.

#### 3.1.5. *Lunidium laculatum* nov. gen. nov. sp.

The vermiform trophozoites are 115–123 µm long and 17 µm wide (*n* = 2), with the ovoid nucleus situated around midpoint, closer to the anterior of the cell (figure 1*e–f*). Both anterior and posterior are narrowing to a blunt point. The cell is translucent and has clearly defined helical epicytic folds running across the surface, making the cell seem to have a cross-hatched pattern. Movement by bending and twisting. Two cells have been sequenced and their assemblies combined, but only one cell is pictured in figure 1. Isolated from the intestinal lumen of a terebellid polychaete *Eupolymnia* sp. (Annelida, Polychaeta, Sedentaria, Terebellida, Terebellidae), found in the reef in Piscadera Bay, in front of the CARMABI research station, Curaçao.

#### 3.1.6. *Lunidium proboscidis* nov. gen. nov. sp.

The trophozoites are vermiform, 110–170 µm long, 18–19 µm wide (*n* = 2), either with a blunt, almost square mucron resembling an elephant’s trunk (figure 1*g,h*) and a posterior ending in a blunt point, or both ends ending in a blunt point. The round nucleus is in the centre of the cell, its widest point. Distinct epicytic folds run longitudinally along the whole length of the cells. The cells move via bending and twisting. Two cells have been sequenced and their assemblies combined, but only one cell is pictured in figure 1. Isolated from the intestinal lumen of a terebellid polychaete *Eupolymnia* sp. (Annelida, Polychaeta, Sedentaria, Terebellida, Terebellidae), found in the reef in Piscadera Bay, in front of the CARMABI research station, Curaçao.

#### 3.1.7. *Lunidium shako* nov. gen. nov. sp.

The vermiform trophozoites measure 108–143 µm in length, 13–19.5 µm in width (*n* = 2), with the anterior ending in a ‘knob’ or hat-like mucron (figure 1*i,j*) and the posterior ending in a blunt point. The oval nucleus is situated roughly at the midpoint of the cell, and distinct epicytic folds run across the whole length of the cell in a helical pattern. The cells move via bending and twisting. Both cells pictured in figure 1 have been sequenced and their assemblies combined. Isolated from the intestinal lumen of a terebellid polychaete *Eupolymnia* sp. (Annelida, Polychaeta, Sedentaria, Terebellida, Terebellidae), found in the reef in Piscadera Bay, in front of the CARMABI research station.

#### 3.1.8. Lunidium melongena

Trophozoites are oval, measuring 116–136 µm in length and 45–64 µm in width (*n* = 3), with a round nucleus in the centre of the cell (figure 1*k*). The mucron is ‘neck-shaped’ and often has host material still attached to it. The cells are commonly found in the coelom of the host, attaching to the intestine from the outside. Three cells were sequenced and coassembled, but only one is pictured in figure 1. The host *Thelepus japonicus* (Annelida, Polychaeta, Sedentaria, Terebellida, Terebellidae) was found in sediment between sea grass at Clover Point, Victoria, BC, Canada.

#### 3.1.9. *Devanium cincinnus* nov. gen. nov. sp.

Trophozoite is vermiform and measures 183 × 9.5 µm, with both anterior and posterior ending in sharp points. Faint longitudinal epicytic folds run along the whole cell (figure 1*l*). The round nucleus is located in the anterior quarter of the cell, and the gregarine moves by bending and twisting. Transcriptome data were derived from the cell imaged. Isolated from a cirratulid bristle worm Cirratulidae sp. (Annelida, Polychaeta, Sedentaria, Terebellida, Cirratulidae) from subtidal surface sediment (8–11 m below sea level) at Hyacinthe Bay, BC, Canada.

#### 3.1.10. *Devanium robustum* nov. gen. nov. sp.

Several trophozoites were found in the intestinal lumen of the same individual of the cirratulid bristle worm *Cirratulus robustus* (Annelida, Polychaeta, Sedentaria, Terebellida, Cirratulidae), with three cells isolated for single-cell transcriptomics. The cells are vermiform with a pointed posterior end, an anterior ending in a ‘knob-like’ mucron (figure 1*m,n*), and range from 104 to 215 µm in length (s.d. = 42.2 µm; *n* = 6) and 10.5–20 µm in width (s.d. = 3.2 µm; *n* = 6). Faint longitudinal epicytic folds are running down the whole cell. The cells move by twisting and bending. The host was found in sediment between sea grass at Clover Point, Victoria, BC, Canada.

#### 3.1.11. *Siedleckia leitoscoloplosis* nov. sp.

The trophozoite is vermiform with a club-like mucron and measures 70–84 µm × 7.5–8 µm (*n* = 2; figure 1*o,p*). Some cell bodies appear ‘layered’ (figure 1*o*), and the mucron has a large vesicle with a granular interior. The cell is translucent and moves by bending and twisting. Two different cells are pictured, but only the cell in figure 1*o* was sequenced. Found in the polychaete *Leitoscoloplos pugettensis* (Annelida, Polychaeta, Sedentaria and Orbiniidae) which was collected from subtidal surface sediment (8–11 m below sea level) at Hyacinthe Bay, BC, Canada.

### 3.2. SSU rDNA phylogenetics

**Figure 2.**
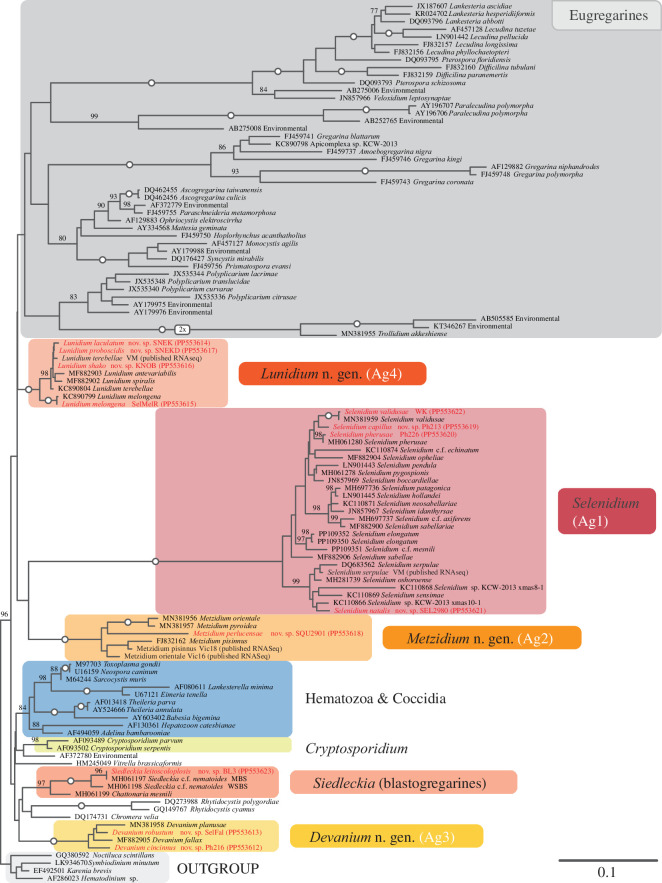
Maximum-likelihood SSU rDNA tree of apicomplexans, generated under the GTR+G model with 1000 non-parametric bootstrap replicates. Sequences from this study are in red and bold. Only support values >75% are shown, and maximally supported internodes (=100%) are marked with a circle.

In our SSU rDNA tree (figure 2), archigregarines do not form a single clade but branch into four distinct lineages: *Selenidium* (lineage Ag1), *Metzidium* nov. gen. (Ag2), *Devanium* nov. gen. (Ag3) and *Lunidium* nov. gen. (Ag4). Each clade is receiving maximum bootstrap support, and blastogregarines are highly supported with 97% (*Siedleckia* and *Chattonaria*). The exact relationships between these five clades remain unclear since the backbone of the topology is completely unsupported (nodes without any reported bootstrap support are ≤50%).

### 3.3. Multigene phylogenetics

**Figure 3.**
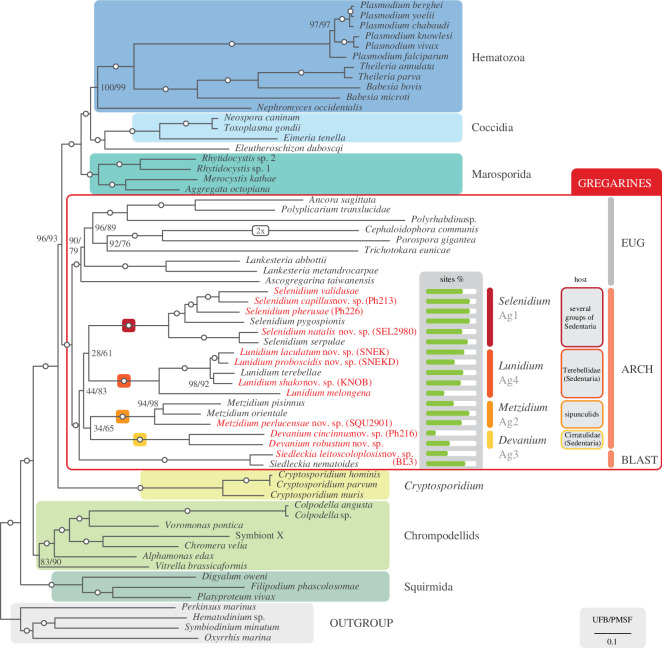
190-gene phylogenomic tree of apicomplexans, generated under LG+C60+F+G with maximum likelihood. Newly added taxa are in red and bold. The first value on internodes is support from 1000 ultrafast bootstraps (UFB), the second from 200 non-parametric bootstraps under LG+C60+F+G+PMSF, and circles mark full bootstrap support with both. The percentage of sites used to estimate the tree is shown for archigregarines and blastogregarines (out of 38 577 amino acid sites).

Our 190-gene phylogenomic tree shows both Apicomplexa and gregarines with full support (figure 3). Eugregarines and the archigregarine+blastogregarine group each form fully supported branches, but the archigregarine clade alone is poorly supported in our main analysis (44% UFB and 83% PMSF) and not at all in many of our additional analyses (22-gene = 18%, FSR = 0–93%, HSR = 0–31%,; electronic supplementary material, figures S2, S5 and S6). As in the SSU rDNA phylogeny, archigregarines separate into four distinct and maximally supported clades representing lineages Ag1-4, but the relationships between them are equally unclear since the archigregarine backbone is unsupported (UFB and PMSF values generally ≤70%). *Siedleckia* forms a fully supported branch that branches sister to the archigregarine group in our main analysis, although this relationship was not strongly supported. In most of our additional analyses *Siedleckia* branches inside archigregarines, sister to *Lunidium* with low to moderate support with UFB (129-gene = 76%, 22-gene = 59%, SR4 = 54%, HSR = 59–98%; electronic supplementary material, figures S1–S3 and S5). A gCF and site-concordance factor analysis (sCF) yielded low support values for the relationships between archigregarine genera and blastogregarines, mirroring the results from bootstrapping (electronic supplementary material, figure S4). gCF values stayed below 10% for any relationships between archi- and blastogregarine clades but ranged from 74% to 94% for individual clades. sCF values followed the same trend, with individual clades ranging from 59% to 93%, but inter-clade relationships hovering around the generally lowest possible value for sCF analyses [43] (32–36%).

## 4. Discussion

### 4.1. Archigregarines and blastogregarines are monophyletic and sister to eugregarines

Archigregarines are pivotal to our understanding of the early evolution of apicomplexans because of their phylogenetic position as sister to all other gregarines, which are together with *Cryptosporidium* the most early diverging lineages of apicomplexans [11]. Only recently have multigene phylogenetics been applied to diverse apicomplexans, and gregarines specifically [9,10,23,44,45], and some gregarine groups remain undersampled, including the archigregarines and blastogregarines. Some recent studies have included multiple species of archigregarines, but a broad sampling reflecting more accurately the diversity of the group has been missing up until now [10,23,45]. In our 190-gene tree, archigregarines and blastogregarines form a maximally supported clade, which in turn branches with eugregarines with full support.

The support for a monophyletic archigregarines is, by contrast, low, which is congruent with a recently published phylogeny that included a much smaller sampling and was missing *Devanium* [23]. It is noteworthy that all archi- and blastogregarine clades are relatively long-branching, but branches connecting the individual clades are short (figure 2; electronic supplementary material, figures S1–S3), which can complicate accurate phylogenetic estimation [42,43]. gCF/sCF yielded the same unresolved topology between clades in this part of the tree, as support values for gCF and sCF were low for the relationships between archigregarine clades but high for the individual clades (electronic supplementary material, figure S4). This probably means that no particular set of genes or sites is driving the observed topology, but that all underlying single-gene data are equally conflicting [42]. Supporting this is also the fact that the smaller and more restrictive 129-gene datasets do not increase support values among the archigregarine–blastogregarine clade significantly (electronic supplementary material, figures S1, 69−76% UFB). We found no evidence of amino-acid composition bias in any of the archigregarines and *Siedleckia* (electronic supplementary material, table S4, generated with PhyloFisher’s aa_comp_calculator.py tool), which can influence and distort phylogenetic inference [46]. Overall, whether blastogregarines branch sister to archigregarines (as in our main phylogeny in figure 3) or alternatively fall within the archigregarines (as in most of our additional analyses in electronic supplementary material, figures S1, S5 and S6) is unclear, as was observed in a previous study [23]. If future analyses confirm blastogregarines branching within archigregarines, the taxonomic descriptions of archigregarines (Archigregarinorida Grassé 1953) could be amended to include blastogregarines rather than keeping them as a separate taxonomic entity of similar level.

It is also possible that the phylogeny may be affected in the future by the discovery and characterization of archigregarine clades that have not yet been discovered and/or sequenced. Here the host-specificity may provide some clues: One species of archigregarine has been described from a hemichordate and two from ascidians [19], yet no molecular sequencing data exists for them, and the taxa in our tree have relatively strong host-specificity. Sequencing archigregarines from host taxa outside those investigated here is perhaps most likely to lead to new subgroups, which in turn may fill in gaps in the topology and help construct a more robust phylogeny, but for now the relationships remain unresolved.

### 4.2. New genera reflect the phylogenetic diversity of archigregarines

Each of the four clades of archigregarines identified in rDNA phylogenies is individually fully supported in phylogenomics, although the exact relationships between them remain unresolved [7,14,17,22]. When identifying these four clades, Paskerova *et al*. stopped short of any wider taxonomic changes, likely due to the lack of data, the fact that only two genes (large and small subunit rDNA) were available at the time, and that the whole group was not resolved in most of these phylogenies [7]. Indeed, the taxonomic confusion of archigregarines and *Selenidium* in particular has been known for several years now and has been noted in the literature repeatedly [7,8,11,18,21–23,47,48]. *Selenidium* is typically unresolved in SSU rDNA phylogenies and has accordingly been described as a ‘paraphyletic stem group’ [7,11,17,47]. Similarly, previous analyses have repeatedly shown that *Selenidium* is hyper-diverse for a genus, encompassing a much wider range of phylotypes than equivalent categories within the apicomplexans or even specifically other gregarine subgroups [7,17,47]. One extreme example of how diverse taxa were lumped within the genus *Selenidium* can be seen in *Platyproteum vivax*, which was initially described as *Selenidium vivax*, but was later transferred to its own novel genus [48], and later found to not even be an apicomplexan but rather a squirmid [49].

With multigene data for all four archigregarine lineages, we now have ample evidence for the hyper-diversity of the genus and reasons to split *Selenidium* based on the four individual well-supported clades in rDNA and multigene phylogenetics (figures 2 and 3), which also correspond to their host-specificity. We propose to retain *Selenidium* to represent lineage Ag1, as it includes the type species *Selenidium pendula* Giard 1884, representing the ‘true’ Selenidiidae or *Selenidium* [7,21]. We also propose to erect three new archigregarine genera to represent the already recognized major subgroups: *Metzidium* nov. gen. for Ag2*, Devanium* nov. gen. for Ag3 and *Lunidium* nov. gen. for Ag4.

The genus *Selenidioides* was established to distinguish *Selenidium* from species that were similar but lacked merogony [19]. Considering proving the absence of a life cycle stage is near impossible, the field has largely abandoned the usage of the genus *Selenidioides* [7,50]. Our SSU rDNA phylogeny reinforces this because several species nominally transferred to *Selenidioides* (*S. axiferens, S. hollandei* and *S. mesnili*) do not form a clade. There is no molecular data for the type species of this disputed genus, *S. caulleryi*, but it is difficult to imagine *Selenidioides* to be a viable genus based on existing data.

Support for each archigregarine lineage and thus distinct genera is strong based on phylogenies and their distinct host specificities. We collated morphological observations from our own and published data and could not find any set of morphological characters that are unique to any genus of archigregarines (electronic supplementary material, table S5). This is not to say none exist, but the current set of data does not show any clear pattern. Additional studies using high-quality light microscopy, scanning electron microscopy (SEM) and transmission electron microscopy (TEM) are needed to explore any potential morphological differences between these genera. For example, the occurrence, structure and organization of epicytic folds, and mucrons can be important morphological markers for archigregarines. Likewise observation of more than a single developmental stage is crucial in further refining taxonomic diagnoses. This is to say our current understanding of the morphological diversity of archigregarines does not reflect the diversity at the molecular level.

The genetic distances between SSU rDNA sequences of different archigregarine genera vary considerably, falling between 65% and 81% similarity (electronic supplementary material, table S6). Genetic distances within genera are lower but still vary considerably, underlining the divergent nature of archigregarine clades. Within *Selenidium* Ag1, for example, SSU rDNA sequences of different species range between 81% and 99% similarity, whereas they range from 79 to 93% in *Metzidium* Ag2.

### 4.3. Host specificity in archigregarine lineages

The four archigregarine subgroups each have a relatively high degree of host-specificity based on current data. Interestingly, the host specificity of *Selenidium* (Ag1) is less strict compared to the other archigregarine lineages—all its hosts are part of Sedentaria (Annelida), a group with over 5000 described species (as per World Polychaete Database, retrieved in May 2024). Ag1 includes specific subgroups of sedentarian hosts that also include hosts of *Devanium* (Ag3) and *Lunidium* (Ag4), and it is possible that a single host species can harbour multiple different archigregarine subgroups (see below). The other three archigregarine lineages are highly host specific. *Metzidium* (Ag2) is the only archigregarine lineage found in peanut worms, annelids that are distantly related to sedentarian polychaetes [48]. *Devanium* (Ag3) and *Lunidium* (Ag4) are to date only found in Cirratulidae and Terebellidae, respectively. *Selenidium* Ag1 also stands out because it has a high degree of both sequence and host diversity and considerably more described species (21 in *Selenidium* Ag1, 4 in *Metzidium* Ag2, 4 in *Devanium* Ag3 and 7 in *Lunidium* Ag4). Interestingly, Ag1 appears to be composed of at least two subclades, perhaps reflecting the larger number of taxa. A larger subclade that includes *Selenidium pendula*, *S. validusae*, *S. pherusae* and *S. capillus*, and a smaller one with *Selenidium serpulae* and *S. natalis* (figure 2). In our multigene tree, both of these clades are maximally supported (figure 3).

Cells of *Selenidium capillus* Ph213 and *Devanium cincinnus* Ph216 were isolated from different individuals of the same host, a cirratulid polychaete. While there is no close hit of the host COI to any sequence in public databases, the COIs from Ph213 and Ph216 are almost identical with 99.4% similarity. While uncommon, several other examples of divergent archigregarine species and even lineages occupying the same host are documented. Two species of *Selenidium* were found in the same species of slime feather duster worm, inhabiting different parts of the gut lumen [31]. *Lunidium terebellae* and *L. melongena* are likewise both found in the spaghetti worm *Thelepus japonicus* [22], with the former inhabiting the gut lumen, whereas the latter is found in the coelom. Christmas tree worms appear to be reservoirs of closely related species of *Selenidium*, with one study finding at least three different species in the intestines of *Spirobranchus giganteus* [8], in addition to *Selenidium natalis* nov. sp. SEL2980 (table 1). The symbiotic polychaete *Spirobranchus* itself represents a species complex [51], with different individuals showing genetic difference, which could lead to a different set of archigregarine symbionts, highlighting the need for increased sampling of marine invertebrates for gregarines.

### 4.4. Novel archigregarine species

Sampling increasingly larger numbers of marine invertebrates also increases the number of gregarines found. While there are over 50 described species of archigregarines [19], molecular data are only available for some, and in most cases, the SSU rRNA gene only. Many already described archigregarines have been sequenced [6,17,21] but also new ones described [7,8,20,22,31,48]. The number of both of these cases is expected to rise. We found several archigregarines in previously unsampled polychaete and sipunculid hosts that fit no existing description. In all of these cases, the SSU rDNA phylogeny shows their phylogenetic distance to related organisms, supporting our decision to assign them as new species.

The three *Lunidium* morphotypes SNEK, KNOB and SNEKD were all isolated from the same host individual, a *Eupolymnia* sp. terebellid worm from Curaçao. We propose new species for all three morphotypes, based on their morphological differences and phylogenetic distances: *Lunidium laculatum* SNEK, *L. shako* KNOB and *L. proboscidis* SNEKD (figures 1–3). The SSU sequence of *L. laculatum* is 93.6% similar to *L. proboscidis* SNEKD and only 90.2% similar to *L. shako*, indicating they should all be considered distinct species. *L. laculatum* has strong helical epicytic folds going across the cell, whereas *L. shako* has a conspicuous ‘knobby’ mucron, and *L. proboscidis* SNEKD lacks both the knobby mucron and does not show the helical striations (figure 1). For each of these *Lunidium* species, we sequenced 2–3 cells separately and co-assembled them based on their morphology and SSU rDNA differences (electronic supplementary material, table S1) further providing support that these are novel species.

It should be noted that our observations of the morphology of all newly described species are relatively limited due to our sampling and imaging approach. In most cases, only a single cell to a handful were observed for a given species, and only at a singular life cycle stage (the trophozoite). This limits our understanding of the morphologies of these species, particularly in terms of cell size and observations of fine details like epicytic folds. Nevertheless, we consider them phylogenetically distinct from already described species and as such they merit novel descriptions, and we believe the standard of evidence is high, with imaging from light microscopy together with megabases of sequencing and thousands of genes.

## 5. Conclusions

Generating single-cell transcriptomes of 12 archigregarine and one blastogregarine species enabled us to generate the most comprehensive multigene dataset of archigregarines to date. While our 190-gene phylogenomic tree and additional phylogenomic analyses did not confidently resolve any branching order among archigregarines, together with existing SSU rDNA phylogeny data, it is now obvious that there are at least four subclades of archigregarines. This highlights the long-noted problem that all archigregarine diversity has been classified as a single genus, *Selenidium*, so we propose three additional genera: *Lunidium*, *Metzidium* and *Devanium*. These genera correspond to lineages identified in multiple published SSU rDNA phylogenetic studies, they are strongly supported in multigene phylogenies and they correspond to strong host-specific patterns. The early-diverging archigregarines and blastogregarines remain a crucial and intriguing group to study, and additional taxon sampling is probably needed to resolve their phylogeny. Another piece of the evolutionary puzzle is whether archigregarines have an apicoplast genome.

## 6. Formal taxonomic description

Apicomplexa (Levine 1970)

Gregarinea (Bütschli 1882, stat. nov. Grassé 1953)

Archigregarinorida (Grassé 1953)

### 6.1. *Lunidium* nov. gen. Lax & Keeling 2024

*Description*: A crown clade comprising *Lunidium terebellae*, *L. antevariabilis*, *L. spiralis*, *L. melongena* and *L. laculatum*. Archigregarines that infect the instestinal lumen of polychaetes of the marine invertebrate family Terebellidae Grube 1851 (Annelida, Sedentaria, Terebellida). This definition corresponds to lineage Ag4, outlined in [14].

*Type species*: *Selenidium terebellae* Ray 1930 (=*L. terebellae*, comb. nov.).

*Etymology*: Named after ‘Luna’, an ancient Roman moon deity, referring to the basionym *Selenidium* (Giard 1884), which was likely named after the moon deity ‘Selene’ in ancient Greek mythology.

*Transfer of existing species to Lunidium*: *L. antevariabilis* (Rueckert & Horák 2017 [18])*, L. melongena* (Wakeman, Heintzelman & Leander 2014 [22])*, L. spiralis* (Rueckert & Horák 2017 [18]) and *L. terebellae* (Ray 1930 [22]) (basionym for all: *Selenidium*; all comb. nov.).

### 6.2. *Metzidium* nov. gen. Lax, Jacko-Reynolds & Keeling 2024

*Description*: A crown clade comprising *Metzidium pisinnus*, *M. orientale*, *M. pyroidea* and *M. perlucensae*. Archigregarines that infect the instestinal lumen of marine invertebrates of the class Sipuncula Rafinesque 1814 (Annelida). This definition corresponds to lineage Ag2, outlined in [14].

*Type species*: *Selenidium pisinnus* (Rueckert & Leander 2009 [48]) (=*M. pisinnus*, comb. nov.).

*Etymology*: Named after ‘Mētztli’ (Nahuatl) or ‘Metzi’, a moon deity in Aztec mythology, referring to the basionym *Selenidium* (Giard 1884), which was likely named after the moon deity ‘Selene’ in ancient Greek mythology.

*Transfer of existing species to* Metzidium: *M. orientale* (Bogolepova 1953)*, M. pisinnus* [48] and *M. pyroidea* (Wakeman 2019) (basionym for all: *Selenidium*; all comb. nov.).

### 6.3. *Devanium* nov. gen. Lax, Park, Na & Keeling 2024

*Description*: A crown clade comprising *Devanium planusae*, *D. fallax*, *D. robustum* and *D. cincinnus*. Archigregarines that infect the instestinal lumen of polychaetes of the marine invertebrate family Cirratulidae Carus 1863 (Annelida, Sedentaria, Terebellida). This definition corresponds to lineage Ag3, outlined in [14].

*Type species*: *Selenidium planusae* Wakeman 2019 (=*D. planusae*, comb. nov.).

*Etymology*: Named after ‘Dziewanna’ (Polish) or ‘Devana’, a Western Slavic moon deity of forests, hunting and the moon. Refers to the basionym. *Selenidium* (Giard 1884), which was likely named after the moon deity ‘Selene’ in ancient Greek mythology.

*Transfer of existing species to* Devanium: *D. fallax* (MacGregor and Thomasson 1965)*, D. planusae* (Wakeman 2019) (basionym for all: *Selenidium*; all comb. nov.).

### 6.4. *Selenidium capillus* nov. sp. Lax, Park & Keeling 2024

Archigregarinorida Grassé 1953

Selenidiidae Brasil 1907

*Selenidium* Giard 1884

*Description*: Trophozoite is vermiform, 216 µm long and 14 µm wide, with a pointed anterior end. The cell is translucent, and the nucleus is situated around the midpoint of the cell. Movement by bending and twisting.

*DNA sequence*: SSU rRNA gene sequence, GenBank accession PP553619.

*Type locality*: Hyacinthe Bay, Quadra Island, British Columbia, Canada.

*Type habitat*: Marine.

*Type host*: Cirratulidae sp. (Annelida, Polychaeta, Sedentaria, Terebellida, Cirratulidae).

*Location in host*: Intestinal lumen.

*Type material*: Cell depicted in figure 1*a*.

*Etymology*: From Latin *‘*capillus’ for ‘hair’, referring to the cirratulid host having tentacles, or ‘hair’.

### 6.5. *Selenidium natalis* nov. sp. Lax, Jacko-Reynolds & Keeling 2024

Archigregarinorida Grassé 1953

Selenidiidae Brasil 1907

*Selenidium* Giard 1884

*Description*: The trophozoite cell is >138 µm (cell ruptured during isolation) and 15 µm wide. The posterior forms into a pointed end, and the ovoid nucleus is situated around the midpoint of the cell. The cell is light brown in colour.

*DNA sequence*: SSU rRNA gene sequence, GenBank accession PP553621.

*Type locality*: Reef in front of CARMABI research station, Curaçao.

*Type habitat*: Marine.

*Type host*: *Spirobranchus giganteus* Pallas, 1766 (Annelida, Polychaeta, Sedentaria, Sabellida and Serpulidae).

*Location in host*: Intestinal lumen.

*Type material*: Cell depicted in figure 1*c*.

*Etymology*: From Latin ‘natalis’ meaning ‘of one’s birth’, commonly used as a meaning of Christmas, referring to the common name for the host, Christmas tree worm.

### 6.6. *Lunidium laculatum* nov. sp. Lax & Keeling 2024

Archigregarinorida Grassé 1953

*Lunidium* Lax & Keeling 2024

*Description*: The vermiform trophozoite is 115–123 µm long and 17 µm wide, with the ovoid nucleus situated around midpoint, closer to the anterior of the cell. Both anterior and posterior are narrowing to a blunt point. The cell is translucent and has clearly defined helical epicytic folds running across the surface, making the cell seem to have a cross-hatched pattern. Movement by bending and twisting.

*DNA sequence*: SSU rRNA gene sequence, GenBank accession PP553614.

*Type locality*: Reef in front of CARMABI research station, Curaçao.

*Type habitat*: Marine.

*Type host: Eupolymnia* sp. Verrill 1900 (Annelida, Polychaeta, Sedentaria, Terebellida, Terebellidae).

*Location in host*: Intestinal lumen.

*Type material*: Cell depicted in figure 1*e,f*.

*Etymology*: From Latin ‘laculatum’ meaning ‘checkered, checked, four-cornered’, referring to epicytic folds in some cells rendering a checkered pattern visible with light microscopy.

*Notes*: SSU rRNA gene sequence 93.6% similar to *L. proboscidis* and 90.2% similar to *L. shako.*

### 6.7. *Lunidium shako* nov. sp. Lax & Keeling 2024

Archigregarinorida Grassé 1953

*Lunidium* Lax & Keeling 2024

*Description*: Ttrophozoites are vermiform and measure 108–143 µm in length, 13–19.5 µm in width. The anteriors ends in a ‘knob’ or hat-like mucron (figure 1*i,j*), and the posterior ends in a blunt point. Distinct epicytic folds run across the whole length of the cell in a helical pattern. The oval nucleus is at the midpoint of the cell. Movement via bending and twisting.

*DNA sequence*: SSU rRNA gene sequence, GenBank accession PP553616.

*Type locality*: Reef in front of CARMABI research station, Curaçao.

*Type habitat*: Marine.

*Type host*: *Eupolymnia* sp. Verrill 1900 (Annelida, Polychaeta, Sedentaria, Terebellida, Terebellidae).

*Location in host*: Intestinal lumen.

*Type material*: Cell depicted in figure 1*i,j*.

*Etymology*: From ‘shako’ (from Hungarian ‘csákó’), a cylindrical cap used in several militaries, which the mucron of this species resembles.

*Notes*: SSU rRNA gene sequence 90.2% similar to *Lunidium laculatum* and 89.4% similar to *Lunidium proboscidis.*

### 6.8. *Lunidium proboscidis* nov. sp. Lax & Keeling 2024

Archigregarinorida Grassé 1953

*Lunidium* Lax & Keeling 2024

*Description*: Trophozoites are vermiform, 110–170 µm long, 18–19 µm wide, with a blunt, almost square mucron resembling an elephant’s trunk, and a widened central part of the cell. The round nucleus sits in the centre of the cell. Epicytic folds run longitudinally along the whole length of the cells. Movement through bending and twisting.

*DNA sequence*: SSU rRNA gene sequence, GenBank accession PP553617.

*Type locality*: Reef in front of CARMABI research station, Curaçao.

*Type habitat*: Marine.

*Type host*: *Eupolymnia* sp. Verrill 1900 (Annelida, Polychaeta, Sedentaria, Terebellida, Terebellidae).

*Location in host*: Intestinal lumen.

*Type material*: Cell depicted in figure 1*g,h*.

*Etymology*: From Latin ‘proboscis’ for snout and the trunk of an elephant because the tip of the mucron resembles an elephant’s trunk.

*Notes*: SSU rRNA gene sequence 93.6% similar to *L. laculatum* and 89.4% similar to *L. shako.*

### 6.9. *Metzidium perlucensae* nov. sp. Lax, Jacko-Reynolds & Keeling 2024

Archigregarinorida Grassé 1953

*Metzidium* Lax, Jacko-Reynolds & Keeling 2024

*Description*: Trophozoite cell is vermiform, 117 µm long and 23 µm wide at its widest point, the posterior ending in a point, the anterior in a rounded blunt point. Fine longitudinal striations cover the whole cell, and the elongated nucleus is situated around the midpoint of the cell, closer to the anterior end. The cell appears light brown.

*DNA sequence*: SSU rRNA gene sequence, GenBank accession PP553618.

*Type locality*: Reef in front of CARMABI research station, Curaçao.

*Type habitat*: Marine.

*Type host*: *Phascolosoma perlucens* Baird, 1868 (Annelida, Sipuncula, Phascolosomatidae).

*Location in host*: Intestinal lumen.

*Type material*: Cell depicted in figure 1*d*.

*Etymology*: From Latin ‘perlucens’ referring to the host *Phascolosoma perlucens* Baird, 1868.

### 6.10. *Devanium robustum* nov. sp. Lax, Na & Keeling 2024

Archigregarinorida Grassé 1953

*Devanium* Lax, Park & Keeling 2024

*Description*: Trophozoite cells measured are 104–215 µm in length and 10.5–20 µm in width. Cells are vermiform with a pointed posterior end and an anterior ending in a capitulum or ‘knob-like’ mucron. Faint longitudinal epicytic folds are running down the whole cell. The cells move by twisting and bending.

*DNA sequence*: SSU rRNA gene sequence, GenBank accession PP553613.

*Type locality*: Clover Point, Victoria, British Columbia, Canada.

*Type habitat*: Marine.

*Type host*: *Cirratulus robustus* Johnson, 1901 (Annelida, Polychaeta, Sedentaria, Terebellida, Cirratulidae).

*Location in host*: Intestinal lumen.

*Type material*: Cell depicted in figure 1*m*.

*Etymology*: From Latin ‘robustum’ meaning ‘hard, solid’, referring to the host species *Cirratulus robustus* Johnson, 1901.

### 6.11. *Devanium cincinnus* nov. sp. Lax, Park & Keeling 2024

Archigregarinorida Grassé 1953

*Devanium* Lax, Park & Keeling 2024

*Description*: Trophozoite is vermiform and measures 183 µm × 9.5 µm, with both anterior and posterior ending in sharp points. Faint longitudinal epicytic folds run along the whole cell. The round nucleus is located in the anterior quarter of the cell. Movement by bending and twisting.

*DNA sequence*: SSU rRNA gene sequence, GenBank accession PP553612.

*Type locality*: Hyacinthe Bay, Quadra Island, British Columbia, Canada.

*Type habitat*: Marine.

*Type host*: Cirratulidae sp. (Annelida, Polychaeta, Sedentaria, Terebellida, Cirratulidae).

*Location in host*: Intestinal lumen.

*Type material*: Cell depicted in figure 1*l*.

*Etymology*: From Latin ‘cincinnus’ meaning ‘lock of hair’, referring to the host having curly tentacles (Cirratulidae sp.).

### 6.12. *Siedleckia leitoscoloplosis* nov. sp. Lax, Park & Keeling 2024

Blastogregarinorina Chatton & Villeneuve 1936

Siedleckiidae Chatton & Villeneuve 1936

*Siedleckia* Caullery & Mesnil 1898

*Description*: The trophozoite is vermiform with a club-like mucron and measures 70–84 µm × 7.5–8 µm. Several ovoid nuclei are stacked on top of each other throughout the cell body, and the mucron has a large vesicle with a granular interior. The cell is translucent and moves by bending and twisting.

*DNA sequence*: SSU rRNA gene sequence, GenBank accession PP553623.

*Type locality*: Hyacinthe Bay, Quadra Island, British Columbia, Canada.

*Type habitat*: Marine.

*Type host*: *Leitoscoloplos pugettensis* Pettibone, 1957 (Annelida, Polychaeta, Sedentaria, Orbiniidae).

*Location in host*: Intestinal lumen.

*Type material*: Cell depicted in figure 1*o*.

*Etymology*: From Greek ‘leitoscoloplos’ referring to the host *Leitoscoloplos pugettensis* Pettibone, 1957.

## Data Availability

Raw transcriptome reads are available under NCBI BioProject accession PRJNA1090553, SSU rRNA gene sequences under accessions PP553612 to PP553623. Transcriptome assemblies, their predicted proteomes, host COI sequences, SSU rRNA gene alignment and tree data, and all multigene alignments and trees (single gene and concatenated) are deposited under Dryad accession [52]. Supplementary material is available online [53].
